# Does an educational video for aneuploidy screening improve informed choice among pregnant women? A randomised controlled trial

**DOI:** 10.1002/pd.6279

**Published:** 2022-12-28

**Authors:** Karen Mei Xian Lim, Celine Lewis, Hung Chew Wong, Glenda Sze Ling Chong, Arundhati Gosavi, Mahesh Arjandas Choolani

**Affiliations:** ^1^ Department of Obstetrics & Gynaecology National University Hospital Singapore Singapore; ^2^ Population, Policy and Practice UCL Great Ormond Street Institute of Child Health London UK; ^3^ London North Genomic Laboratory Hub Great Ormond Street Hospital London UK; ^4^ Yong Loo Lin School of Medicine National University of Singapore Singapore Singapore

## Abstract

**Background:**

Poor knowledge and the lack of deliberation have been cited as reasons for women making uninformed choices about aneuploidy screening. Adequate pre‐test counselling is of particular importance where non‐invasive prenatal screening (NIPS) is being increasingly offered as a primary screening test.

**Design:**

Women attending the antenatal clinic with a singleton pregnancy below 14 weeks were randomised to receive routine counselling or the intervention—a 16‐min educational video on aneuploidy screening before their consult. The primary outcome, rate of informed choice, was assessed using an adapted multidimensional measure of informed choice questionnaire, where informed choice was defined as good knowledge and value‐consistent behaviour. Secondary outcomes included informed choice with deliberation, decisional conflict and anxiety.

**Results:**

Two hundred and eighty‐six women were recruited. 69.8% of women in the intervention group made an informed choice compared with 53.6% in the control group (Risk Ratio [RR] 1.30, *p* = 0.014). A significantly higher number of women in the intervention group had good knowledge compared to controls (81% vs. 60.9%; RR 1.33, *p* = 0.001). Decisional conflict did not differ between groups, but women in the intervention group had higher anxiety scores (*p* < 0.001).

**Conclusion:**

The study intervention was effective in helping women make informed choice. Qualitative studies to determine the reason for increased anxiety are needed.

**Trial registration:**

Trial registry: ClinicalTrials.gov; Identifier: NCT05492981.

## Key points

What is already known about this topic?

- Non‐invasive prenatal screening (NIPS) is increasingly being offered as a first‐line screening option for aneuploidy.
- Women risk making uninformed choices due to routinisation of testing.
- Pre‐test counselling should be directed towards helping women make informed choice.

What does this study add?

- A large number of women make uninformed choices due to poor knowledge about testing.
- An educational video is able to help women make informed choices by increasing knowledge, especially in settings where NIPS is offered as a first‐line option.

## 1 INTRODUCTION

Options for aneuploidy screening have increased in the last decade. Following the discovery of cell‐free DNA and the introduction of non‐invasive prenatal screening (NIPS) as an option for aneuploidy screening,^1^ the rate of invasive tests performed in women who are at increased risk of having a child with an aneuploidy has greatly reduced in number.^2^, ^3^, ^4^, ^5^ NIPS, which was mostly offered as part of contingent screening or only to high‐risk women, is now increasingly being offered to all women regardless of background risk in countries, such as Australia, the United States of America, and certain parts of Europe.^6^, ^7^ This is following recommendations from the American College of Obstetricians and Gynaecologists in 2020.^8^ NIPS is offered as a primary screening test option to all women in Singapore.^9^ This is, however, self‐funded, in contrast to countries, such as Belgium where NIPS is publicly funded.^10^ Despite its relatively high‐cost ranging from Singapore Dollar (SGD) 1100 to SGD 2500,^11^ the high sensitivity of NIPS at 99% as well as the non‐invasive nature of the test still makes it an attractive and acceptable option to pregnant women.^3^ This results in an increased uptake of testing, but also risks possible routinisation of testing,^12^ where women choose to test because it is routinely offered and available without really having considered the pros, cons and implications of the results on their pregnancies.^13^ This has been thought to result either from a lack of deliberation and/or adequate pre‐test counselling. Such routinisation of unique genetic information obtained from screening has been described^14^ and is of particular concern when a complex test, such as NIPS, can be performed by a simple blood test, potentially leading to uninformed choices.^10^, ^13^

A well‐accepted definition of informed choice is ‘one that is based on relevant knowledge, consistent with the decision‐maker's values and behaviourally implemented’.^15^, ^16^ Studies have shown that many women are making uninformed choices when it comes to aneuploidy screening^17^, ^18^ with rates of informed choice as low as 37% in certain populations.^19^ The psychological effects of uninformed choices are known, such as greater decisional conflict and regret,^20^ and decreased patient satisfaction.^21^ Several interventions have been designed to help women make informed choices about aneuploidy screening,^22^, ^23^, ^24^, ^25^, ^26^ most of which were trialled in settings where the combined first‐trimester screen (FTS) was the primary test being offered for screening in low‐risk women. Newer studies have investigated methods to improve knowledge about NIPS^27^ and informed choice in a setting where NIPS is offered as part of contingent screening.^25^ However, no studies have been performed on improving informed choice in the era where NIPS is offered as a primary screening option. We developed an educational video on the current options for aneuploidy screening covering what the test entails as well as possible results of each test and their implications. We hypothesised that this intervention, in addition to a routine consult with the obstetrician, would be able to improve the rates of informed choice amongst women compared to standard counselling by the clinician alone. If found to be effective, this intervention could act as a useful adjunct to the clinical consult, which can be implemented as part of all routine antenatal consults where aneuploidy screening is discussed with women.

## 2 METHODS

### 2.1 Trial design

This randomised controlled trial enroled women attending the antenatal clinic for their routine visits at National University Hospital Singapore between July 2021 and February 2022. The trial obtained ethics approval by the Domain Specific Review Board (Reference number: 2020/01123) and was registered on ClinicalTrials.gov (Identifier: NCT05492981).

### 2.2 Patients

Eligibility criteria included women who were aged 21 years or older, who were English speaking and who had a viable singleton pregnancy below 14 weeks gestation. Additionally, women must not have had any prior discussion with a clinician regarding aneuploidy screening for their current pregnancy. Written information leaflets on FTS and NIPS were available in clinic waiting rooms and used during antenatal consults by doctors when counselling women about aneuploidy screening. In Singapore, all women are routinely offered the option of FTS or NIPS as a primary screening test, which is self‐funded. The cost of FTS ranges from SGD 130 to SGD 270, compared with the cost of NIPS, which ranges from SGD 1100 to SGD 2500.^11^

### 2.3 Procedures

Eligible participants were referred by doctors running the antenatal clinic after subjects were confirmed to have a viable singleton pregnancy on ultrasound. Consent was obtained by members of the study team and subjects were randomised to receive the intervention or control. Women were randomised to receive the intervention or standard care using computer‐generated randomisation. Randomisation allocation was placed in sealed opaque sequentially numbered envelopes. The study team member recruiting subjects was blinded to the randomisation sequence. Due to the nature of the intervention, blinding of subjects was not possible. Women randomised to the intervention were asked to watch a 16‐min educational video in a separate room. They then resumed their consult with the referring doctor to clarify any questions and to inform them of which screening option they decided on, if any. Women in the control group were counselled by the referring doctor regarding the aneuploidy screening options of FTS or NIPS and provided with information about the procedures involved for each test, their detection rates, cost, and possible results of testing. The use of a written information leaflet was available to doctors as an adjunct to providing this information. The length of the consult ranged anywhere from 5 to 15 min depending on whether the subject had any questions pertaining to pregnancy or aneuploidy screening.

The intervention was a 16‐min educational video created by the study investigators, which provided information about Down syndrome, diagnostic tests and screening tests, and is available via the following link: https://drive.google.com/file/d/1dSYFmpwpWZ9HupLBFbmGVDXUpTF11f2C/view?usp=drivesdk. The screening tests covered in the video included the triple test, the combined FTS and NIPS. Information about how and when each of the tests were performed, their detection rates and possible results and their interpretations were covered in the video. The video was viewed by several healthcare providers and experts in the field of prenatal screening before the commencement of this study to ensure that the content was appropriate and adequate. The intervention was viewed by subjects on a portable tablet in a quiet room in the antenatal clinic. Subjects were then invited to ask their doctor any questions that they may have had about the video during the clinic consult which followed. This clinic consult ranged from 5 to 15 min depending on whether the subject had any questions pertaining to pregnancy or aneuploidy screening.

Following the consultation with the doctor, subjects in both the intervention and control group were asked to complete a questionnaire, which assessed the primary outcome measure of informed choice. The questionnaire also assessed background demographics of patients, including age, ethnicity, educational background, parity, whether they were religious, and whether they had performed aneuploidy screening in their previous pregnancies.

### 2.4 Outcomes

The primary outcome was the percentage of women who made an informed choice in the intervention group compared with control. The definition of informed choice was one that was made with good knowledge and value‐consistent behaviour. Secondary outcomes included informed choice with deliberation, knowledge, value‐consistent behaviour, anxiety and decisional conflict. Subgroup analyses were performed in women aged 35 years or older, those with a university degree, women who were religious, women who were parous, and women who had performed Down syndrome screening in their previous pregnancies.

The assessment of informed choice was performed using a self‐administered questionnaire at the end of the clinic visit. A widely used measure of informed choice is the Multidimensional Measure of Informed Choice (MMIC), which was developed for women undergoing aneuploidy screening in pregnancy, assessing knowledge and attitudes towards the combined FTS.^15^, ^28^ This was further modified and validated as an instrument for assessing informed choice in a setting where both FTS and NIPS were offered.^25^, ^29^ The following components were assessed to determine if a subject had made an informed choice: knowledge, attitudes and uptake.

An informed choice was deemed to have been made if the subject had good knowledge and chose a testing option which was consistent with her attitude towards that test. Knowledge was assessed using the knowledge scale of the MMIC, which comprised 19 validated questions that covered important aspects of FTS, NIPS, and invasive testing that were deemed essential for an informed choice.^25^, ^30^ Total knowledge scores of more than or equal to 12, representing >60% correct responses, were determined to indicate sufficient knowledge.^25^ The attitude scale of the MMIC assessed whether patients' attitudes towards the three different testing options (combined FTS, NIPS and invasive testing) were positive or negative. As only FTS and NIPS were offered as options for aneuploidy screening, attitudes related to these were used to assess informed decision‐making in this study. Scores between 0 and 6 indicated a positive attitude towards having the test, scores between 7 and 13 indicated a neutral attitude, and scores between 14 and 20 indicated a negative attitude towards having the test.^31^ Women were then asked to input which method of aneuploidy testing they had decided on. This decision was confirmed by the checking of medical records to ascertain which test women eventually chose. Women were determined to have made a behaviourally consistent choice if they had a positive attitude towards the testing option that they eventually chose or if they had a negative attitude towards any of the testing options and did not choose to test. Women who had neutral attitudes to any of the tests that they had chosen were excluded from the analysis.^29^, ^31^ An informed choice was made if a subject had good knowledge, had a positive attitude towards the combined FTS and chose that as a screening option. Similarly, women were deemed to have made an informed choice if they had good knowledge, had a negative attitude towards all modes of testing, and chose not to perform any aneuploidy screening. Women were deemed to have made an uninformed choice if they had poor knowledge or if their eventual behaviour (choice of testing) was not consistent with their attitude towards that test.

A deliberation scale was added to the original MMIC in a previous study, as whether women deliberated their decision was thought to be an important component of informed decision making.^29^, ^32^ The deliberation scale consisted of six five‐point Likert items, which assessed if participants had weighed the pros and cons of testing, and was included in the version of the MMIC used in this study. Possible scores ranged from 0 to 24. Individuals who had deliberation scores less than 13, which are the midpoint of the scale, were considered to have deliberated their decisions.^29^, ^32^ Examples of statements in the deliberation scale included ‘I have imagined how I would feel if I did not have prenatal testing’, and ‘I have tried to think through the consequences of not accepting prenatal testing’. The results were presented as an informed choice with and without deliberation.

Measures to assess decisional conflict and anxiety were included in a previous study by Beulen et al., which investigated the use of a decision‐making aid in a setting where NIPS was offered as part of contingent screening,^25^ and these scales were used in this study.

Decisional conflict was measured using a validated Decisional Conflict Scale,^33^, ^34^ which assessed how certain women felt about their decision and whether they perceived that they had autonomy in making their decision. Scores above 37.5 were associated with uncertainty about implementation of the decision.

Anxiety was assessed using the Spielberger State Trait Anxiety Inventory (STAI‐6) short form, which consisted of six items.^35^ A mean of the total scores was compared between the control and intervention groups. Higher scores correlated with higher anxiety.

### 2.5 Statistical analysis

We determined that a 15% improvement in informed choice in the intervention group was clinically significant based on a similarly designed study, on a background rate of informed choice in the control group of 70%.^25^ We calculated that 121 subjects were required to detect this with 80% power at a 5% level of significance. Accounting for a 15% attrition rate, 143 subjects in each arm were calculated to be the target sample size, giving a total of 286 subjects.

A Chi‐Square test was used to analyse the test uptake between the intervention and control groups. Robust Poisson regression models were used to assess the rates of informed choice, good knowledge, and value‐consistent behaviour between the intervention and control groups. Subgroup analyses were also performed for the primary outcome of informed choice. The subgroups were age, education level, religion and whether women had aneuploidy screening in their previous pregnancies. A robust Poisson regression model was also used to evaluate the proportion of women who had significant decisional conflict (scores above 37.5) between those who made informed and uninformed choices. Comparisons of the proportion of women who had significant decisional conflict between the intervention and control groups were also carried out before and after adjusting for informed choice using robust Poisson regression. Median regression was used to evaluate whether there was a significant difference in the decisional conflict score between the intervention and control groups. A 2‐sample *t*‐test was used to analyse whether there was a significant difference in the STAI‐6 total scores and deliberation total scores between the intervention and control groups. Robust Poisson regression models were also used to determine whether there was a significant difference in the rates of deliberation (yes/no) between the intervention and control groups overall, and also among those who chose to test (acceptors) and those who did not (decliners). Statistical significance was set at 5%. The statistical analyses were performed using SAS version 9.4 (SAS Institute, Inc; Cary, NC, USA).

## 3 RESULTS

**FIGURE 1:**
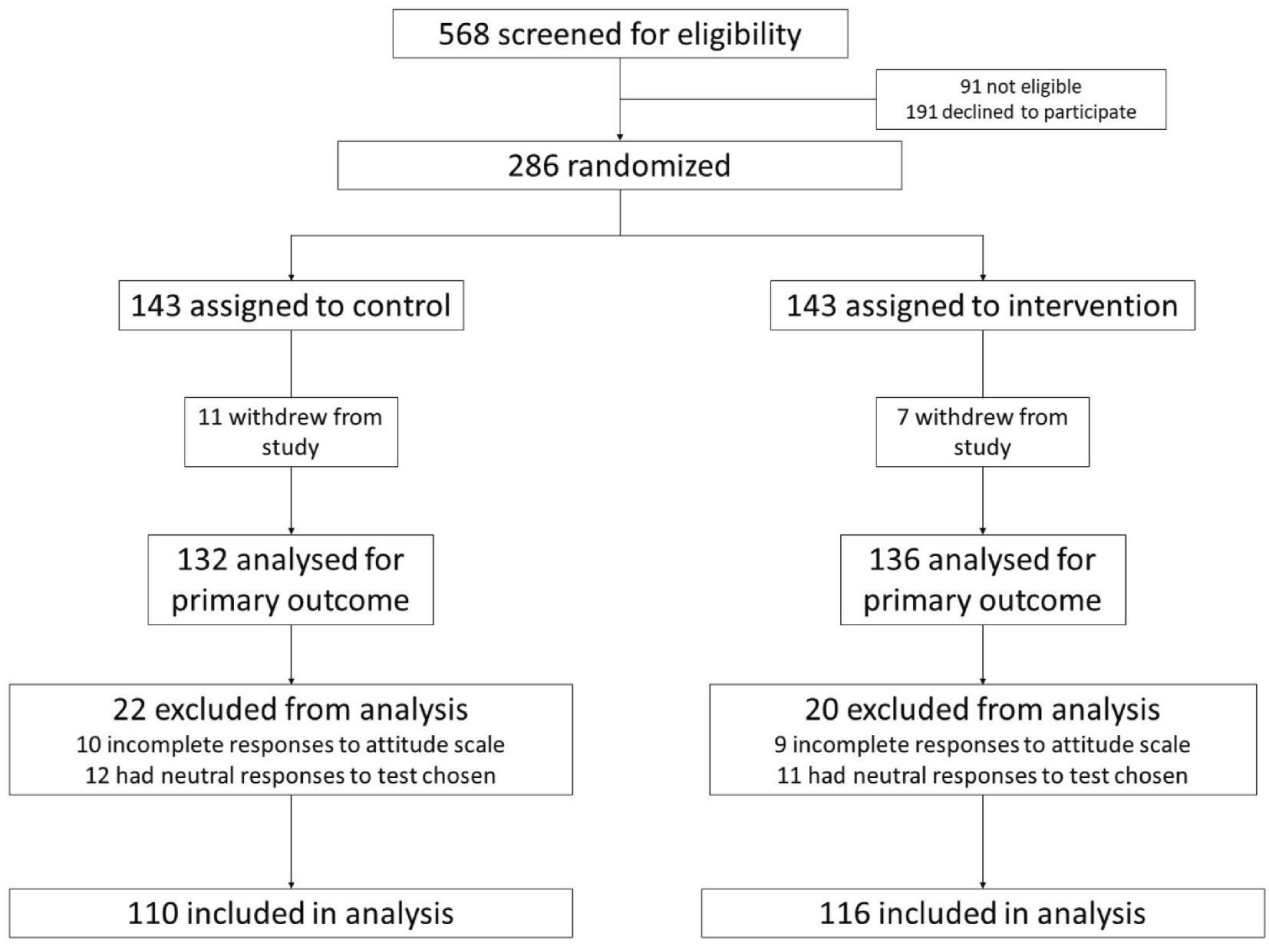
CONSORT flow diagram

Overall, 568 women were screened for eligibility between July 2021 and February 2022; 286 women consented to participate in the trial, 143 were randomised to receive the intervention and 143 were randomised to the control group (Figure 1). In total, 7 and 11 subjects withdrew from the study in the intervention and control group, respectively. Twenty subjects in the intervention group and 22 subjects in the control group were excluded from the final analysis due to incomplete responses on the questionnaire (*n* = 19) or neutral responses to the test option that they had chosen (*n* = 23). This left 110 and 116 subjects in the final analysis in the control and intervention groups, respectively.

Baseline characteristics were largely similar between the intervention and control groups and are presented in Table 1. Majority of women had a degree level of education or above and considered themselves religious.

**TABLE 1:** Baseline characteristics

	Control (*N* = 132)	Intervention (*N* = 136)
Maternal age (years)		
Mean (SD)	29.5 (3.8)	30.2 (4.1)
Age ≥35	11 (8.3%)	21 (15.4%)
Education level		
No qualification	1 (1%)	0 (0%)
GCSE or O level	20 (15%)	14 (10%)
GCE, A level or similar	3 (2%)	10 (7%)
Diploma	33 (25%)	49 (36%)
Degree level or above	75 (57%)	63 (46%)
Ethnicity		
Chinese	51 (38%)	50 (38%)
Malay	63 (48%)	73 (53%)
Indian	9 (7%)	7 (5%)
Other	9 (7%)	6 (4%)
Religious faith		
Yes	111 (84%)	122 (90%)
No	21 (16%)	14 (10%)
Parity		
Parous	47 (35.6%)	56 (41%)
Nulliparous	85 (64.4%)	80 (59%)
Aneuploidy screening in previous pregnancy		
Yes	22 (46.8%)	27 (47%)
No	24 (51.1%)	28 (49%)
Not sure	1 (2.1%)	2 (4%)

Abbreviations: GCE: General Certificate of Education; GCSE: General Certificate of Secondary Education.

In total, 69.8% of women in the intervention group made an informed choice compared with 53.6% in the control group (Risk Ratio, RR, 1.30 [95% Confidence Interval, CI, 1.05–1.61] *p* = 0.014). A significantly higher number of women in the intervention group had good knowledge compared to controls (81% vs. 60.9%, RR 1.33 [95% CI 1.12–1.58] *p* = 0.001). There were no significant differences in value‐consistent behaviour between the groups (RR 1.01 [95% CI 0.90 to −1.13] *p* = 0.86).

Of the 51 participants who made an uninformed choice in the control group, poor knowledge was found to be the cause in 64.7% of participants, value‐inconsistent behaviour in 15.7% and both poor knowledge and value‐inconsistent behaviour in 19.6%. Of the 35 patients who made an uninformed choice in the intervention group, poor knowledge was found to be the cause in 48.5% of participants, value‐inconsistent behaviour in 37.1% and both poor knowledge and value‐inconsistent behaviour in 14.3%.

Regarding test uptake, 37.7% of women in the control group chose not to test compared with 41.5% in the intervention group. The breakdown of test uptake is shown in Table 2.

**TABLE 2:** Test uptake

Test	Control (*n* = 130)^a^	Intervention (*n* = 135)^a^	*p*‐Value
Combined first‐trimester screen	56 (43.0%)	39 (28.9%)	0.016
Non‐invasive prenatal screening	25 (19.2%)	40 (29.6%)	0.05
Did not test	49 (37.7%)	56 (41.5%)	0.53

^a^ Two subjects in the control group and one subject in the intervention group did not make a decision.

When subgroup analyses were performed for the primary outcome, informed choice was significantly higher in the intervention group in women aged <35 years (RR 1.35, [95% CI 1.08–1.70], *p* = 0.009). In women aged 35 years and above, the intervention did not show a significant increase in informed choice (RR 0.95, [95% CI 0.57–1.60], *p* = 0.85). A significant increase in informed choice in the intervention group was also noted in the subgroups of participants with an education level of Diploma or below as well as women who were religious; however, previously having had aneuploidy screening in a prior pregnancy was not found to be a significant factor (Table 3).

**TABLE 3:** Subgroup analyses

Subgroup	Control, % (*n*/*N*)	Intervention, % (*n*/*N*)	*p*‐Value
Women aged <35 years	52.0% (52/100)	70.4% (69/98)	0.009
Women aged 35 years or older	70.0% (7/10)	66.7% (12/18)	0.854
Degree holders	71.2% (47/66)	84.7% (50/59)	0.069
Non‐degree holders	27.3% (12/44)	54.4% (31/57)	0.012
Religious	53.2% (50/94)	68.0% (70/103)	0.028
Not religious	56.3% (9/16)	84.6% (11/13)	0.103
Had screened in previous pregnancies	64.7% (11/17)	73.9% (17/23)	0.541
Did not screen in previous pregnancies	47.8% (11/23)	56% (14/25)	0.574

### 3.1 Secondary outcomes

When deliberation was included as a criterion for making an informed choice, rates of informed choice were no different between the control and intervention groups (48.2% vs. 56.9%, RR 1.18 [95% CI 0.92–1.52], *p* = 0.19). There was no statistically significant difference between the proportion of women who deliberated their decisions in the intervention and the control groups. Mean deliberation scores and rates of deliberation among test acceptors and decliners are presented in Table 4.

**TABLE 4:** Secondary outcomes

	Control	Intervention	*p*‐Value
Proportion of all subjects who deliberated, % (*n*/*N*)	89.1% (98/110)	82.8% (96/116)	0.172
Proportion of test acceptors who deliberated, % (*n*/*N*)	93.8% (61/65)	87.3% (55/63)	0.209
Proportion of test decliners who deliberated, % (*n*/*N*)	82.2% (37/45)	77.4% (41/53)	0.548
Mean deliberation score, mean (SD)	7.19 (5.21)	7.31 (5.15)	0.794
Decisional conflict total score, median (minimum–maximum)	25 (0–75)	25 (0–90)	1
Proportion of all subjects who had decisional conflict^a^	25.5% (28/110)	16.4% (19/116)	0.097
			0.219^b^
Anxiety total score by STAI‐6, mean (SD)	38.94 (8.31)	42.44 (5.15)	<0.001

Abbreviation: STAI‐6, State Trait Anxiety Inventory.

^a^ Scores above 37.5, which was associated with uncertainty about implementation of decision.

^b^ After adjusting for informed choice.

Fewer women who made informed choices experienced decisional conflict compared to women who made uninformed choices (30.2% vs. 15%, RR 0.50 [95% CI 0.30–0.83]; *p* = 0.007). However, there were no statistically significant differences in the number of women who experienced decisional conflict between the control and intervention groups before (*p* = 0.097) and after adjusting for whether or not they had made informed choices (*p* = 0.219).

Mean anxiety scores were noted to be higher in the intervention group (*p* < 0.001) with a mean difference of 3.50 (95% CI 1.68–5.33) (Table 4).

## 4 DISCUSSION

Our study suggests that the use of an educational video could help women make informed choices about aneuploidy screening. In previous studies, poor knowledge was often cited as a reason for uninformed choice.^23^, ^36^ Women in the intervention group had higher knowledge scores compared with control, suggesting that improving knowledge may be a possible mechanism for improving informed choice. The findings from this study are particularly clinically relevant as NIPS increasingly becomes offered as a first‐line screening test among low‐risk women.

A systematic review of the literature regarding informed choice showed that women preferred to learn about NIPS from their clinician.^10^ However, many reported dissatisfaction with the information that they had been provided with and felt that the information was inadequate to empower informed choice.^10^, ^37^, ^38^, ^39^ Additionally, the review reported that women often felt that consultations were too short to address the concerns and questions that they had about NIPS.^40^ The intervention we designed for this study aimed to provide detailed information about aneuploidy screening as well as act as an adjunct to the clinical consult. This allowed women to spend the time with their clinician clarifying questions about the information provided by the video as well as allowed standardisation of the information provided to patients. The results of our study are similar to that demonstrated by Mulla et al., where a 15‐min educational video designed by the study team was effective in improving knowledge in women below 35 years of age. However, in their study, women aged 35 years and older were additionally counselled by a genetic counsellor as part of standard care, leaving little room for knowledge improvement with the intervention.^27^ In our study, subgroup analyses showed that informed choice was higher in women aged below 35 years who received the intervention, but not in the participants aged 35 years and above. This may possibly due to the fact that women aged 35 years and above already perceive themselves to be at a higher risk of aneuploidy and may have sought information on screening prior to the consult.

We additionally found that the rate of informed choice in the control group of 53.6% was relatively low compared with a study performed in Netherlands where NIPS is also offered as a primary screening option, which reported up to 75% of women making informed choices in their population.^41^ However, a different questionnaire comprising of seven questions was used to measure knowledge in that study and hence comparisons are limited. Poor knowledge was the reason that an uninformed choice was made in the majority of the women in the control group in our study. Higher rates of informed choice were noted in participants without university degrees who received the intervention compared to those that did not. However, this difference was not seen in university degree holders. This could be due to a higher baseline knowledge of aneuploidy screening in women with higher education, which has previously been reported.^29^ Additionally, women who considered themselves to be religious also made more informed choices with the intervention. As numbers in each subgroup are small, the conclusions that can be drawn from these subgroup analyses are limited, but highlight a subset of antenatal women who might benefit most from such interventions.

When deliberation was included as a criterion for making an informed choice, the intervention did not appear to result in a higher proportion of women making informed choices. Previous studies, which had included deliberation as a criterion for informed decision‐making, reported that the lack of deliberation was the main reason for uninformed choices.^29^, ^32^ Whether this finding is due to the failure of a questionnaire to capture deliberation adequately, or whether women are truly not deliberating their decisions can only be ascertained by qualitative interviews, which were not performed in this study. Regardless, this finding highlights the need for any intervention targeted at improving informed choice to not only increase knowledge but help women consider the pros and cons of testing as well as the implications of their results. This may be achieved in practice through decision aids where prompts are incorporated into an interactive version of the intervention, which act as value‐ and preference‐clarification exercises. Such heuristic‐based deliberation tools and decision aids have been shown to be well received by women when making healthcare decisions in the prenatal setting.^25^, ^42^, ^43^

The psychological effects of uninformed choice have been described, such as increased decisional conflict,^17^, ^20^, ^28^ which has been associated with later decisional regret.^44^ This was consistent with the findings from our study that less women who made informed choices experienced significant decisional conflict compared with women who made uninformed choices as a whole. However, actual decisional conflict scores did not differ significantly between the intervention and control, despite there being higher rates of informed choice. This could be due to higher decisional conflict scores among women who made uninformed choices despite receiving the intervention, resulting in a higher‐than‐expected mean score in the intervention group. Additionally, mean anxiety scores appeared higher in the intervention group. This finding is consistent with that of another study, which suggested that providing women with more information might result in increased anxiety.^17^ A longitudinal study to measure anxiety scores before and after women receive their test results if they chose to test would be helpful in determining the true effect of the intervention on anxiety.

A notable finding of this study was that women in the intervention group appeared more likely to choose NIPS, while women in the control group appeared more likely to choose FTS as a screening option. Such a finding highlights the potential influence that the pre‐test counselling has on eventual test choice, in line with concerns about routinisation of testing. The observed difference in test choice between the two arms may be attributable to the possibility that the difference in limitations of FTS and NIPS was better explained by the intervention than the clinician's counselling, resulting in participants in the control arm simply choosing the cheaper test option. Qualitative interviews with subjects regarding their decisions and which component of the counselling influenced their decision would be valuable. Additionally, a survey of healthcare providers on how confident they felt in explaining the characteristics, advantages and limitations of each test would prove useful in determining causality.

The strength of our study is that it is a randomised controlled trial performed in a setting, which offers both FTS and NIPS as primary screening options. While a significant amount of research has been performed on interventions aimed at improving informed choice with regard to prenatal screening, few studies were performed in settings where NIPS is offered as a primary screening option.^18^, ^45^ As the clinical applications of non‐invasive prenatal testing continue to expand, an effective method of providing information to increase patient knowledge and improve informed choice will become increasingly important in the clinical setting. Additionally, the intervention is easily implementable in other clinical settings and does not require additional resource as the video is now freely available online.

Although a randomised controlled trial, there are several limitations in this study. The first is that it lacks qualitative data. Structured interviews with participants would be helpful in tailoring the contents of the video to suit their information needs. Secondly, although participants in the intervention group had higher knowledge scores compared to controls after having received the intervention, the design of the study does not prove that the intervention improved knowledge scores from baseline. Randomisation of participants aimed to reduce the possibility of selection bias, but baseline knowledge scores were not measured to prove that this was not different between groups. Thirdly, comparison of the length of clinic consult times would have been useful in determining whether the intervention helped to reduce consult times with the clinician as demonstrated in a similar study regarding informed consent for hysterectomy.^46^ It would also help to exclude a potential confounder that participants in the intervention group had a longer time to consider their options, resulting in them having made more informed choice, although deliberation scores did not seem to differ between the groups. However, measurement of consult times was not performed in this study due to logistical limitations. Fourthly, this study was only performed in the pre‐testing setting and does not give insight into the effect of the intervention on how women received their test results if they had tested, and whether it resulted in higher or lower satisfaction and decisional regret. Lastly, this study only included English‐speaking women as the video was only available in English. This may have resulted in a selection bias, where only a particular demographic of women was included. Future plans to translate the video into the main languages spoken in Singapore exist to reach a wider population.

## 5 CONCLUSION

This study adds to the current literature of interventions targeted at improving informed choice in women making decisions regarding prenatal testing. It is of particular relevance in the current era of NIPS, which is an area that is constantly expanding. Widespread implementation of the intervention has the potential to help women make informed choices. Future research should look into how the intervention could be modified to improve deliberation rates amongst women so as to avoid routinisation of testing with care not to increase anxiety amongst women.

## CONFLICT OF INTEREST

The author(s) report(s) no conflict of interest.

## Data Availability

Individual level data (excluding identifiers) will be made available on request. Non‐identifiable participant responses and clinical metadata will be available. The institutional review board‐approved study protocol will also be made available on request. Data will be available from the date of publication to 5 years after enrolment of the final subject. Non‐identifiable data will be shared with other researchers on request to facilitate reproducibility or for inclusion in a systematic review.

## References

[1] Lo YM , Corbetta N , Chamberlain PF , et al. Presence of fetal DNA in maternal plasma and serum. Lancet. 1997;350(9076):485‐487. 10.1016/s0140-6736(97)02174-0 9274585

[2] Hui L , Muggli EE , Halliday JL . Population‐based trends in prenatal screening and diagnosis for aneuploidy: a retrospective analysis of 38 years of state‐wide data. BJOG. 2016;123(1):90‐97. 10.1111/1471-0528.13488 26108969

[3] Gil MM , Accurti V , Santacruz B , Plana MN , Nicolaides KH . Analysis of cell‐free DNA in maternal blood in screening for aneuploidies: updated meta‐analysis. Ultrasound Obstet Gynecol. 2017;50(3):302‐314. 10.1002/uog.17484 28397325

[4] Horsting JM , Dlouhy SR , Hanson K , Quaid K , Bai S , Hines KA . Genetic counselors' experience with cell‐free fetal DNA testing as a prenatal screening option for aneuploidy. J Genet Couns. 2014;23(3):377‐400. 10.1007/s10897-013-9673-4 24352524

[5] Chetty S , Garabedian MJ , Norton ME . Uptake of noninvasive prenatal testing (NIPT) in women following positive aneuploidy screening. Prenat Diagn. 2013;33(6):542‐546. 10.1002/pd.4125 23592525

[6] Allyse M , Minear MA , Berson E , et al. Non‐invasive prenatal testing: a review of international implementation and challenges. Int J Women's Health. 2015;7:113‐126. 10.2147/ijwh.s67124 25653560 PMC4303457

[7] Ravitsky V , Roy MC , Haidar H , et al. The emergence and global spread of noninvasive prenatal testing. Annu Rev Genom Hum Genet. 2021;22(1):309‐338. 10.1146/annurev-genom-083118-015053 33848430

[8] Rose NC , Kaimal AJ , Dugoff L , Norton ME , American College of Obstetricians and Gynecologists . Screening for fetal chromosomal abnormalities: ACOG practice bulletin, number 226. Obstet Gynecol. 2020;136(4):e48‐e69. 10.1097/aog.0000000000004084 32804883

[9] Yang L , Tan WC . Prenatal screening in the era of non‐invasive prenatal testing: a nationwide cross‐sectional survey of obstetrician knowledge, attitudes and clinical practice. BMC Pregnancy Childbirth. 2020;20(1):579. 10.1186/s12884-020-03279-y 33004009 PMC7528474

[10] Cernat A , De Freitas C , Majid U , Trivedi F , Higgins C , Vanstone M . Facilitating informed choice about non‐invasive prenatal testing (NIPT): a systematic review and qualitative meta‐synthesis of women's experiences. BMC Pregnancy Childbirth. 2019;19(1):27. 10.1186/s12884-018-2168-4 30642270 PMC6332899

[11] Barrett AN , Advani HV , Chitty LS , et al. Evaluation of preferences of women and healthcare professionals in Singapore for implementation of noninvasive prenatal testing for Down syndrome. Singapore Med J. 2017;58(6):298‐310. 10.11622/smedj.2016114 27357315 PMC5474525

[12] Vanstone M , Cernat A , Majid U , Trivedi F , De Freitas C . Perspectives of pregnant people and clinicians on noninvasive prenatal testing: a systematic review and qualitative meta‐synthesis. Ont Health Technol Assess Ser. 2019;19(5):1‐38. PMC6398533 30838086

[13] Press N , Browner CH . Why women say yes to prenatal diagnosis. Soc Sci Med. 1997;45(7):979‐989. 10.1016/s0277-9536(97)00011-7 9257391

[14] Foster MW , Royal CD , Sharp RR . The routinisation of genomics and genetics: implications for ethical practices. J Med Ethics. 2006;32(11):635‐638. 10.1136/jme.2005.013532 17074820 PMC2563298

[15] Marteau TM , Dormandy E , Michie S . A measure of informed choice. Health Expect. 2001;4(2):99‐108. 10.1046/j.1369-6513.2001.00140.x 11359540 PMC5060053

[16] O'Connor A , O'Brien‐Pallas L . Decisional conflict. In: Nursing diagnosis and intervention. Vol 2. The C.V. Mosby Company; 1989.

[17] Green JM , Hewison J , Bekker HL , Bryant LD , Cuckle HS . Psychosocial aspects of genetic screening of pregnant women and newborns: a systematic review. Health Technol Assess. 2004;8(33). iii, ix‐x, 1‐109. 10.3310/hta8330 15298822

[18] Ames AG , Metcalfe SA , Dalton Archibald A , Duncan RE , Emery J . Measuring informed choice in population‐based reproductive genetic screening: a systematic review. Eur J Hum Genet. 2015;23(1):8‐21. 10.1038/ejhg.2014.89 24848746 PMC4266751

[19] Rowe HJ , Fisher JR , Quinlivan JA . Are pregnant Australian women well informed about prenatal genetic screening? A systematic investigation using the multidimensional measure of informed choice. Aust N Z J Obstet Gynaecol. 2006;46(5):433‐439. 10.1111/j.1479-828x.2006.00630.x 16953859

[20] Bekker HL , Hewison J , Thornton JG . Applying decision analysis to facilitate informed decision making about prenatal diagnosis for Down syndrome: a randomised controlled trial. Prenat Diagn. 2004;24(4):265‐275. 10.1002/pd.851 15065100

[21] van den Berg M , Timmermans DR , Ten Kate LP , van Vugt JM , van der Wal G . Are pregnant women making informed choices about prenatal screening? Genet Med. 2005;7(5):332‐338. 10.1097/01.gim.0000162876.65555.ab 15915085

[22] Björklund U , Marsk A , Levin C , Öhman SG . Audiovisual information affects informed choice and experience of information in antenatal Down syndrome screening‐‐a randomized controlled trial. Patient Educ Couns. 2012;86(3):390‐395. 10.1016/j.pec.2011.07.004 21807474

[23] Dormandy E , Michie S , Hooper R , Marteau TM . Informed choice in antenatal Down syndrome screening: a cluster‐randomised trial of combined versus separate visit testing. Patient Educ Couns. 2006;61(1):56‐64. 10.1016/j.pec.2005.02.006 16533677

[24] Nagle C , Gunn J , Bell R , et al. Use of a decision aid for prenatal testing of fetal abnormalities to improve women's informed decision making: a cluster randomised controlled trial [ISRCTN22532458]. BJOG. 2008;115(3):339‐347. 10.1111/j.1471-0528.2007.01576.x 18190370

[25] Beulen L , van den Berg M , Faas BH , et al. The effect of a decision aid on informed decision‐making in the era of non‐invasive prenatal testing: a randomised controlled trial. Eur J Hum Genet. 2016;24(10):1409‐1416. 10.1038/ejhg.2016.39 27189020 PMC5027684

[26] Graham W , Smith P , Kamal A , Fitzmaurice A , Smith N , Hamilton N . Randomised controlled trial comparing effectiveness of touch screen system with leaflet for providing women with information on prenatal tests. BMJ. 2000;320(7228):155‐160. 10.1136/bmj.320.7228.155 10634736 PMC27263

[27] Mulla BM , Chang OH , Modest AM , Hacker MR , Marchand KF , O'Brien KE . Improving patient knowledge of aneuploidy testing using an educational video: a randomized controlled trial. Obstet Gynecol. 2018;132(2):445‐452. 10.1097/aog.0000000000002742 29995739 PMC6059992

[28] Michie S , Dormandy E , Marteau TM . The multi‐dimensional measure of informed choice: a validation study. Patient Educ Couns. 2002;48(1):87‐91. 10.1016/s0738-3991(02)00089-7 12220754

[29] Lewis C , Hill M , Skirton H , Chitty LS . Development and validation of a measure of informed choice for women undergoing non‐invasive prenatal testing for aneuploidy. Eur J Hum Genet. 2016;24(6):809‐816. 10.1038/ejhg.2015.207 26508572 PMC4867447

[30] Schoonen HM , van Agt HM , Essink‐Bot ML , Wildschut HI , Steegers EA , de Koning HJ . Informed decision‐making in prenatal screening for Down's syndrome: what knowledge is relevant? Patient Educ Couns. 2011;84(2):265‐270. 10.1016/j.pec.2010.07.037 20800415

[31] van den Berg M , Timmermans DR , Kleinveld JH , Garcia E , van Vugt JM , van der Wal G . Accepting or declining the offer of prenatal screening for congenital defects: test uptake and women's reasons. Prenat Diagn. 2005;25(1):84‐90. 10.1002/pd.1090 15662690

[32] van den Berg M , Timmermans DR , ten Kate LP , van Vugt JM , van der Wal G . Informed decision making in the context of prenatal screening. Patient Educ Couns. 2006;63(1–2):110‐117. 10.1016/j.pec.2005.09.007 16242899

[33] O'Connor AM . Validation of a decisional conflict scale. Med Decis Mak. 1995;15(1):25‐30. 10.1177/0272989x9501500105 7898294

[34] Koedoot N , Molenaar S , Oosterveld P , et al. The decisional conflict scale: further validation in two samples of Dutch oncology patients. Patient Educ Couns. 2001;45(3):187‐193. 10.1016/s0738-3991(01)00120-3 11722854

[35] Marteau TM , Bekker H . The development of a six‐item short‐form of the state scale of the Spielberger State‐Trait Anxiety Inventory (STAI). Br J Clin Psychol. 1992;31(3):301‐306. 10.1111/j.2044-8260.1992.tb00997.x 1393159

[36] Gourounti K , Sandall J . Do pregnant women in Greece make informed choices about antenatal screening for Down's syndrome? A questionnaire survey. Midwifery. 2008;24(2):153‐162. 10.1016/j.midw.2006.09.001 17316936

[37] Lewis C , Hill M , Skirton H , Chitty LS . Fetal sex determination using cell‐free fetal DNA: service users' experiences of and preferences for service delivery. Prenat Diagn. 2012;32(8):735‐741. 10.1002/pd.3893 22573474

[38] Agatisa PK , Mercer MB , Leek AC , Smith MB , Philipson E , Farrell RM . A first look at women's perspectives on noninvasive prenatal testing to detect sex chromosome aneuploidies and microdeletion syndromes. Prenat Diagn. 2015;35(7):692‐698. 10.1002/pd.4594 25800864

[39] Floyd E , Allyse MA , Michie M . Spanish‐ and English‐speaking pregnant women's views on cfDNA and other prenatal screening: practical and ethical reflections. J Genet Couns. 2016;25(5):965‐977. 10.1007/s10897-015-9928-3 26739840 PMC4936962

[40] Lau JY , Yi H , Ahmed S . Decision‐making for non‐invasive prenatal testing for Down syndrome: Hong Kong Chinese women's preferences for individual vs relational autonomy. Clin Genet. 2016;89(5):550‐556. 10.1111/cge.12743 26864268

[41] van der Meij KRM , Njio A , Martin L , et al. Routinization of prenatal screening with the non‐invasive prenatal test: pregnant women's perspectives. Eur J Hum Genet. 2021. 10.1038/s41431-021-00940-8 PMC9177612 34385671

[42] Durand MA , Wegwarth O , Boivin J , Elwyn G . Design and usability of heuristic‐based deliberation tools for women facing amniocentesis. Health Expect. 2012;15(1):32‐48. 10.1111/j.1369-7625.2010.00651.x 21241434 PMC5060608

[43] Yu L , Yang S , Zhang C , et al. Decision aids for prenatal testing: a systematic review and meta‐analysis. J Adv Nurs. 2021;77(10):3964‐3979. 10.1111/jan.14875 33942356

[44] Hartwig TS , Borregaard Miltoft C , Malmgren CI , Tabor A , Jørgensen FS . High risk‐what's next? A survey study on decisional conflict, regret, and satisfaction among high‐risk pregnant women making choices about further prenatal testing for fetal aneuploidy. Prenat Diagn. 2019;39(8):635‐642. 10.1002/pd.5476 31083781

[45] Skjøth MM , Draborg E , Pedersen CD , Hansen HP , Lamont RF , Jørgensen JS . Providing information about prenatal screening for Down syndrome: a systematic review. Acta Obstet Gynecol Scand. 2015;94(2):125‐132. 10.1111/aogs.12543 25412186

[46] Pallett AC , Nguyen BT , Klein NM , Phippen N , Miller CR , Barnett JC . A randomized controlled trial to determine whether a video presentation improves informed consent for hysterectomy. Am J Obstet Gynecol. 2018;219(3):277.e1‐277.e7. 10.1016/j.ajog.2018.06.016 29959929

